# Supplementary material for: Splice-Junction-Based Mapping of Alternative Isoforms in the Human Proteome
Source: Cell Rep. Author manuscript; Available in PMC 2020 Jan 15. (PMC6961840; doi:10.1016/j.celrep.2019.11.026)
Supplement: 3 [file NIHMS1546469-supplement-3.zip › DF2/PXD0010154/Spleen-64-P22105-MGPLSVVIVTVAR.pdf]

A

Predicted sequence disorder and sequence features of  P22105

Peptide:  MGPLSVVIVTVAR Junction:  sp|P22105|TENX_HUMAN|ENSG00000168477|SE2|39943|chr6|32070414|32072298|−2|r15|T1  TrNovel: TRUE

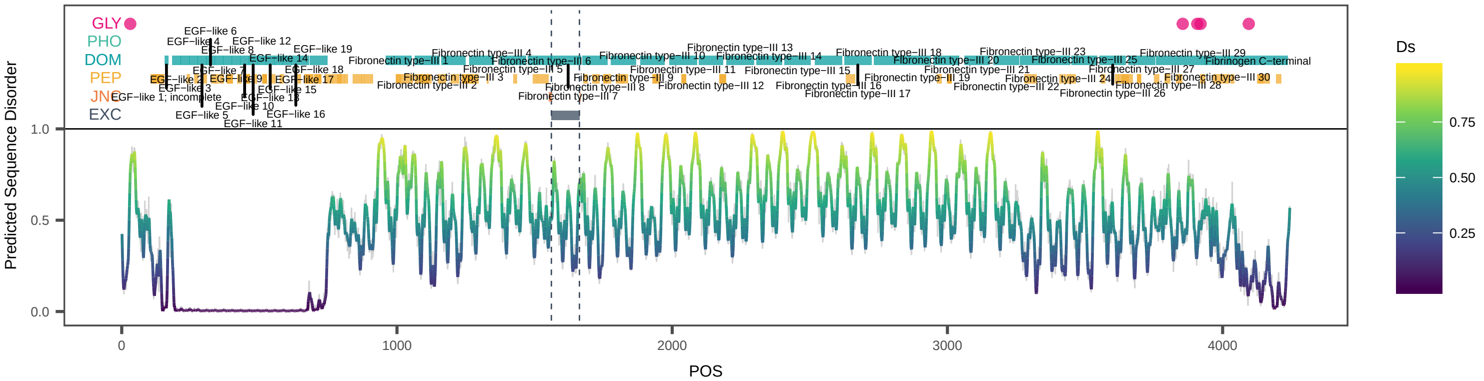


B

Distribution of sequence disorder in excised
vs. mapped and non−excised regions of protein

M−W P−value vs. mapped:  0.000608 vs. non−excised:  0.774

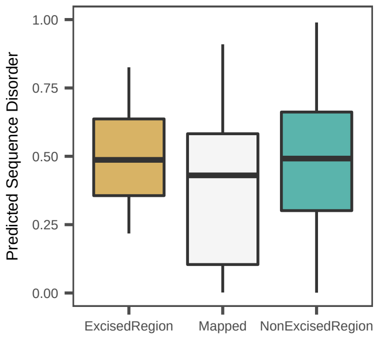


C
